# Clinicopathologic and Molecular Characteristics of Gastrointestinal MiNENs

**DOI:** 10.3389/fonc.2021.709097

**Published:** 2021-08-04

**Authors:** Min-Kyung Yeo, Nara Yoon, Go Eun Bae

**Affiliations:** ^1^Department of Pathology, Chungnam National University School of Medicine, Daejeon, South Korea; ^2^Departments of Pathology, Incheon St. Mary’s Hospital, College of Medicine, The Catholic University of Korea, Incheon, South Korea

**Keywords:** ATRX, gastrointestinal, neoplasms, neuroendocrine, molecular, sequence, pathology

## Abstract

**Background:**

A mixed neuroendocrine–non-neuroendocrine neoplasm (MiNEN) is a recently defined entity that comprises a neuroendocrine tumor (NEN) component and a non-neuroendocrine tumor (nNEN) component. As MiNEN is a recently defined entity, its molecular nature is not well known. Here, we evaluated the clinicopathologic and molecular characteristics of gastrointestinal (GI) MiNENs.

**Methods:**

We performed a genomic analysis of 31 samples from 12 GI MiNEN cases using next-generation sequencing. We examined the primary NEN and nNEN components, as well as the metastatic NENs and nNENs. The relationships between the clinical tumor features (component, location, and grade) and their molecular characteristics were examined.

**Results:**

The 12 MiNENs included in the study were found in the stomach (n=10), distal rectum (n=1), and anus (n=1). Primary MiNENs that had NENs as the major component showed a worse clinical outcome than those that had nNENs as the major component. All distant metastatic tumors originating from MiNENs were NENs. In addition, NENs generally carried 1.5 times more gene mutations and copy number variations than nNENs. The ATRX gene deletion and TP53 gene mutation were the most common variants in both components of GI MiNENs.

**Conclusions:**

We have revealed the detailed clinicopathologic and molecular findings with distinguishable alterations of GI MiNENs. To our knowledge, this is the first study to report the ATRX gene deletion in GI MiNENs. The molecular characteristics of GI MiNENs could provide clues to the pathogenic origin and progression of GI MiNENs.

## Introduction

A mixed neuroendocrine–non-neuroendocrine neoplasm (MiNEN) is a very rare neoplasm that consists of two morphologically and immunohistochemically distinct components, a neuroendocrine tumor (NEN) component and a non-neuroendocrine tumor (nNEN) component, with each component constituting more than 30% of the neoplasm (1). Most MiNENs are known to arise in the gastrointestinal (GI) tract and typically present as small or large cell neuroendocrine carcinomas (SCNEC or LCNEC, respectively) in the case of NEN and adenocarcinomas in the case of nNEN (1, 2).

The mechanism underlying the tumorigenesis of MiNENs is unclear; however, three theories have been proposed: (i) the neuroendocrine and non-neuroendocrine components merge after arising independently from separate progenitor cells, (ii) the two components derive from a common pluripotent stem cell progenitor that undergoes bi-phenotypic differentiation during carcinogenesis, and (iii) the two components originate from common monoclonal cells, but neuroendocrine differentiation occurs in a non-neuroendocrine phenotype following the accumulation of molecular aberrations (3–5). Several studies have attempted to characterize the main genetic and epigenetic aberrations underlying MiNENs, and have sought to identify both the biological similarities between the two components and potential therapeutic targets. However, current knowledge of MiNENs is based on case reports and small retrospective studies in which the pathologic and molecular characteristics of MiNENs are not well defined (5).

In the GI tract, nNENs are usually located superficially near to mucosa, whereas NENs are located at the invasive front of the MiNEN in a deeper area of the GI tract. Therefore, we hypothesized that a superficial nNEN may develop into an NEN *via* the accumulation of molecular aberrations that induce a transitional morphologic change. Here, we used next-generation sequencing (NGS) to examine the primary NEN and nNEN components of GI MiNENs, as well as their nodal and distant metastatic tumors. We compared the NGS results with the clinicopathologic characteristics of the GI MiNENs, including the histologic tumor components, location, and grade.

## Materials and Methods

### Cases and Tissue Samples

From January 2012 to December 2019, 12 patients who underwent surgical excision at Chungnam National University Hospital, Daejeon, South Korea. were diagnosed with MiNEN tumors. Two licensed pathologists (Bae and Yeo) reviewed and confirmed all cases) according to the 5th edition of the WHO Classification of Digestive System Tumors (1). Clinical and pathological data, including the age of cases at initial diagnosis, type of surgical treatment, duration of follow-up, histologic subtype, depth of invasion, lymphovascular and perineural invasion, lymph node metastasis, presence of local recurrence and/or distant metastasis were obtained from the electronic medical record system, imaging studies, and pathology reports. This study was approved by the Institutional Review Board (IRB) at the Chungnam National University Hospital, Daejeon, South Korea (2019–11–043), and was performed in compliance with the Declaration of Helsinki. Because the study was retrospective, a waiver of consent was approved by the IRB.

### Next-Generation Sequencing and Data Analysis

A total of 31 formalin-fixed paraffin embedded (FFPE) tissues from 12 patients were available for DNA/RNA extraction. Among them, three samples from one patient failed to yield adequate quality of DNA. Therefore, a total of 28 samples from 11 patients, including 11 pairs of NENs and nNENs and six metastatic tumors were successfully examined. DNA and RNA were isolated from 10 µm sections of tumor FFPE tissue samples using a sterile 26-gauge needle and the RecoverAll™ Multi-Sample RNA/DNA Isolation Workflow (Ambion, Austin, TX, USA), according to the manufacturer’s instructions. Each primary NEN, nNEN, and metastatic tumor component was obtained by manual microdissection, and DNA and RNA were extracted for library preparation. For each case, normal control tissue was also dissected from an adjacent non-malignant region. DNA and RNA were quantified using a Qubit 2.0 fluorometer (Thermo Fisher Scientific, Waltham, MA, USA). The libraries were generated from 10 ng of DNA and RNA per sample using the IonAmpliSeq™ Kit for Chef DL8, the Ion 540 Chef kit, and the Ion S5™ Chef system (all Thermo Fisher Scientific), according to the manufacturer’s instructions.

Sequencing was performed using the Ion S5 sequencer and Ion 540 chips (Thermo Fisher Scientific), according to the manufacturer’s instructions. Sequencing data analysis was performed using Torrent Suite version 5.10.2 and Ion Reporter version 5.6 (Thermo Fisher Scientific), as well as the commercial pan-cancer Oncomine Comprehensive Assay version 3. The Oncomine panel enables analysis of variations in 161 genes, including 86 mutational hotspot oncogenes and 48 full-length tumor suppressor genes, (all exons); copy number variations (CNV) in 47 genes and fusion drivers in 51 genes (**Supplementary Table 1**). The workflow was created by adding custom hotspots Browser Extensible Data (BED) file to report mutations of interest (MOIs) and a custom CNV baseline (described in the next paragraph) using the manufacturer’s default workflow as previously described (6).

ANNOVAR software (http://www.openbioinformatics.org/annovar/) was used for functional annotation of the identified single nucleotide polymorphisms (SNPs) to investigate their genomic locations and variations (7). To eliminate error artifacts, sequence data were confirmed visually using the Integrative Genomics Viewer. This workflow could identify SNVs and indels with a variant allele fraction as low as 1%. Based on the results of a feasibility study, the variant allele fraction threshold was established at 3%.

Somatic SNVs/indels that passed filtering in gain-of-function genes (oncogenes) were considered gain-of-function when they occurred at the predefined hotspot residues targeted by the Oncomine panel. Somatic variants in a loss-of-function gene were considered loss-of-function when deleterious (nonsense or frame shifting) changes occurred at a pre-defined hotspot residue. Copy number analysis was performed using the copy number module within the previously mentioned Ion Reporter system workflow. If copy numbers of target genes were four or more, they were considered as amplifications. Additionally, if copy numbers of target genes were less than one, they were considered as deletions. Somatic CNVs were considered for potential actionability analysis when they were concordant with predicted alteration (amplification or deletion) from the Oncomine analysis, as described above. Somatic gene fusions were considered for actionability analysis when they represented known gene fusions from the Mitelman database (National Cancer Institute, Bethesda, MD, USA) or Oncomine analysis, or if they involved known 3′ or 5′ drivers with novel partners. These prioritized variants were then associated with potential actionability using the Oncomine database. For each patient, the “most actionable” alteration was identified based on the following criteria: (i) variants referenced in Food and Drug Administration (FDA) drug labels, (ii) variants referenced in National Comprehensive Cancer Network (NCCN) treatment guidelines for the patient’s cancer type, (iii) variants referenced in an NCCN guideline for another cancer type, and (iv) variants referenced as inclusion criteria in a clinical trial. Actionable variants were identified by manual curation of FDA labels and NCCN guidelines, as well as by keyword searches and manual curation of clinical trial records in the TrialTrove database (6). The genetic variants identified were interpreted by a board-certified pathologist (Yoon) and categorized as “pathogenic”, “likely pathogenic”, “variant of unknown significance”, “presumed benign,” or “benign”, based on their clinical significance according to ClinVar-indexed variants (National Center for Biotechnology Information, USA) (8). When assessing the mutation frequencies of individual genes, “pathogenic” and “presumed pathogenic” were counted as mutations, whereas “benign” “presumed benign”, and “variants of unknown significance” were excluded.

## Results

### Clinicopathologic Characteristics of the MiNEN Cases

The male-to-female ratio was 11:1 and the median age of the subjects was 66.4 years (range, 50–81 years). The follow-up interval ranged from 2 to 71 months with a median of 4 months. Ten cases were located in the stomach, with one case in the lower rectum and one case in the anus. Gastric MiNEN cases presented frequently in mid and lower body (50%) and antrum (30%) locations. The sizes of the tumors ranged from 1.7 to 7 cm (median, 3.84 cm). The pathologic tumor stages (pTs) were classified as pT1/T2 (n=6, 50%) and pT3/T4 (n=6, 50%), and the stage groups were classified as I/II (n=3, 25%) and III/IV (n=9, 75%). In 12 cases, regional lymph node metastasis was detected in nine (75.0%) and distant metastasis (only liver) in three (25%). Two cases displayed liver metastasis at the time of diagnosis and another case presented delayed liver metastasis during follow-up. **Table 1** summarizes the clinicopathologic characteristics of the 12 GI MiNEN cases included in the study.

**Table 1:** Clinicopathologic characteristics of the 12 gastrointestinal MiNEN cases included in the study.

Characteristics	Number of patients (%)
Age at diagnosis	
<60 years	3 (25.0)
≥60 years	9 (75.0)
Sex	
male	11 (91.7)
female	1 (8.3)
Primary tumor site	
stomach	10 (83.3)
rectum, anus	2 (16.7)
Size	
<5 cm	9 (75.0)
≥5 cm	3 (25.0)
Lymph node metastasis	
absent	3 (25.0)
present	9 (75.0)
Distant metastasis	
absent	9 (75.0)
present	3 (25.0)
Pathologic T stage	
T1/T2	6 (50.0)
T3/T4	6 (50.0)
Stage group	
I/II	3 (25.0)
III/IV	9 (75.0)
Adjuvant therapy	
no	7 (58.3)
yes	5 (41.7)
Histology of primary nNEN	
Adenocarcinoma	10 (83.3)
tubular type	8
mucinous type	2
Tubular adenoma, high-grade	2 (16.7)
Histology of primary NEN	
neuroendocrine tumor grade 3	3 (25.0)
SCNEC	2 (16.7)
LCNEC	7 (58.3)
Histology of nodal metastatic tumor	
nNEN	3 (37.5)
NEN	5 (62.5)
Major tumor component (>50%) of primary tumor	
nNEN	6 (50)
NEN	6 (50)

LCNEC, large cell neuroendocrine carcinoma; NEN, neuroendocrine tumor component; NETG3, neuroendocrine tumor grade3; SCNEC, small cell neuroendocrine carcinoma.

**Figure 1:**
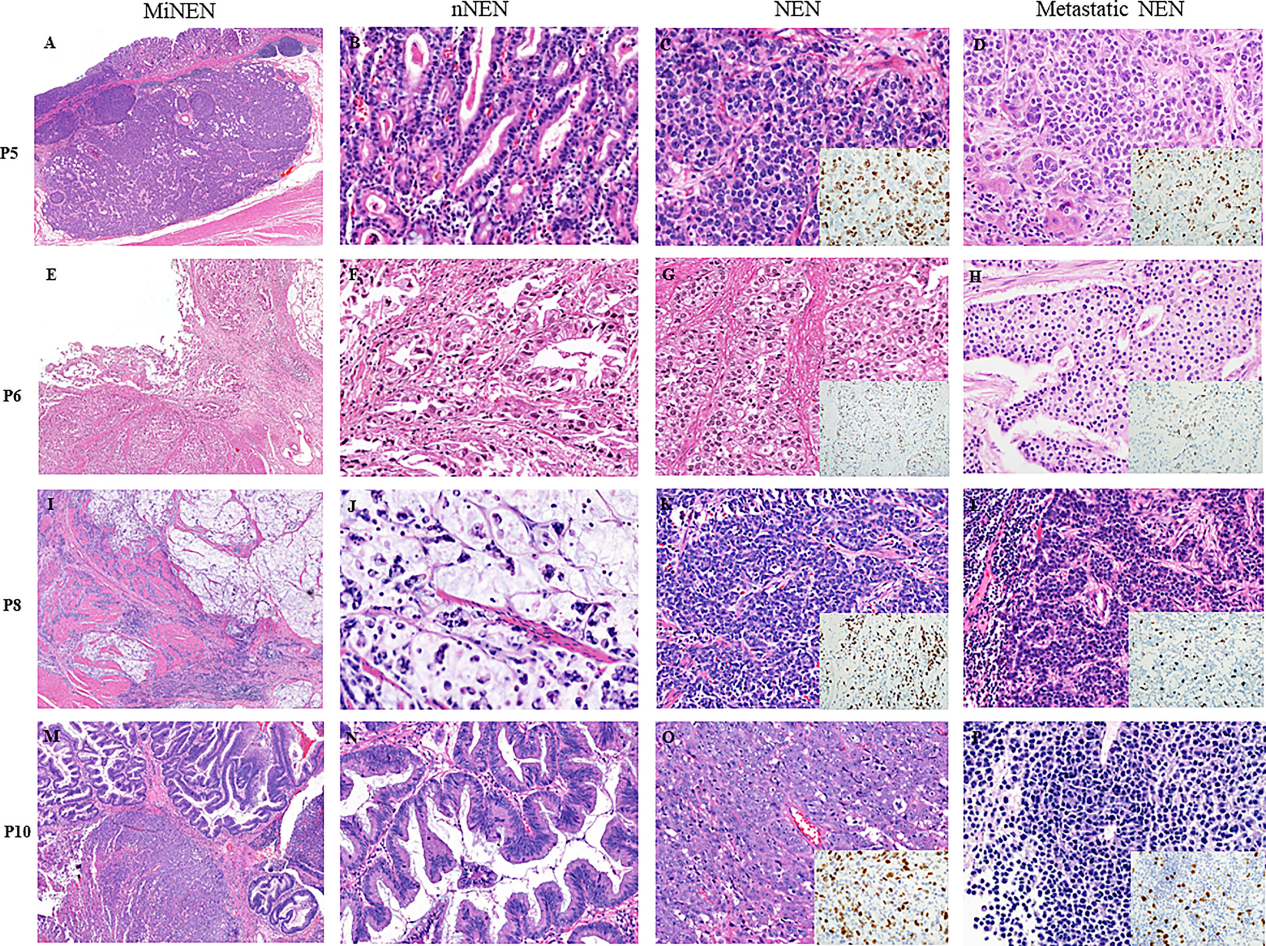
Microscopic features of four representative cases of MiNENs. **(A–D)** An early gastric MiNEN (pT1) from patient 5. **(B–D)** show a mucosal well-differentiated adenocarcinoma, submucosal large cell neuroendocrine carcinoma (LCNEC), and hepatic metastatic LCNEC. **(E–H)** showing a gastric MiNEN from patient 6. **(F–H)** show a tubular adenocarcinoma and a neuroendocrine tumor grade 3(NETG3) and nodal metastatic NETG3. **(I–L)** show a gastric MiNEN from patient 8. **(J–L)** show a high-grade mucinous adenocarcinoma, a small cell neuroendocrine carcinoma (SCNEC), and a nodal metastatic SCNEC. **(M–P)** show a locally excised anal MiNEN from patient 10. **(N–P)** show a high-grade serrated adenoma, a LCNEC, and a hepatic metastatic neuroendocrine tumor. **(P)** show less aggressive neuroendocrine tumor cells without prominent nucleoli and necrosis than those of the original large cell neuroendocrine carcinoma. Insets: ki-67 immunohistochemical stains of the each NENs. All tissue sections were stained with hematoxylin and eosin. NEN, neuroendocrine tumor component; NEN, neuroendocrine tumor component; mNEN, metastatic neuroendocrine tumor.

**Figure 1** shows representative histologic features of four MiNENs. In the 12 cases examined, NENs were commonly located in the deeper portion of the GI tract, whereas nNENs were located superficially. The NENs were histologically classified as LCNEC (n=7, 58.3%), neuroendocrine tumor grade 3 (NETG3) (n=3, 25%), or SCNEC (n=2, 16.7%). The nNENs were classified as adenocarcinomas (tubular and mucinous types) (n=10, 83.3%) or high-grade adenomas (tubular and serrated types) (n=2, 16.7%), the latter of which were only identified in anorectal areas. The eight lymph nodal metastatic tumors that were pathologically confirmed consisted of five NENs and three nNENs; the origin of each metastatic tumor matched the major tumor component (>50% tumor volume) of the primary MiNEN, and all liver metastatic tumors were NENs.

### Molecular Comparison of the Tumor Components of GI MiNENs

A total of 28 samples from 11 patients, including 11 pairs of NENs and nNENs and six metastatic tumors, were examined successfully using NGS. The results of the sequencing analysis are shown in **Tables 2** and **3**. The most common genomic variations seen in the GI MiNENs were a TP53 gene mutation and an ATRX gene deletion. Across all 28 samples, pathogenic missense mutations were detected in four genes: TP53 (n=18 samples, 64.3%), ARID1A (n=3, 10.7%), PIK3CA (n=3, 10.7%), and CTNNB1 (n=3, 10.7%). CNVs were observed frequently at various sites; copy number gain was detected for the CCNE1 (n=6, 21.4%), MYC (n=4, 14.3%), TERT (n=3, 10.7%), FGF19 (n=1, 3.6%), CCND1 (n=1, 3.6%), FGF3 (n=1, 3.6%), RICTOR (n=1, 3.6%), KRAS (n=1, 3.6%), AKT (n=1, 3.6%), and FGFR1 (n=1, 3.6%) genes, and copy number loss was detected for the ATRX (n=10, 35.7%), CDKN2A (n=6, 21.4%), CDKN2B (n=6, 21.4%), RB1 (n=3, 10.7%), NBN (n=1, 3.6%), RAD50 (n=1, 3.6%), and FANCD2 (n=1, 3.6%) genes.

**Table 2:** The mutational landscape of the 11 primary gastrointestinal MiNENs.

Patient	Site	Histology (% of component)	Ki-67 index of NEN (%)	Gene	Code change	AA change	Allelic frequency (%)	CNV (copy number)
1	stomach	ACA T (40)		TP53	c.401T>G	p.Phe134Cys	29.42	
				CCNE1				Amplification (6.11)
		NET G3 (60)	30	TP53	c.401T>G	p.Phe134Cys	35.92	
				CCNE1				Amplification (18.86)
2	stomach	ACA T (70)		TP53	c.844C>T	p.Arg282Trp	29.43	
		SCNEC (30)	60	TP53	c.844C>T	p.Arg282Trp	42.40	
				RB1				Deletion (0.66)
3	stomach	ACA T (60)		ARID1A	c.1435C>T	p.Gln479Ter	30.62	
				TP53	c.527G>A	p.Cys176Tyr	22.64	
		LCNEC (40)	50	ARID1A	c.1435C>T	p.Gln479Ter	55.65	
				TP53	c.527G>A	p.Cys176Tyr	56.29	
				CCND1				Amplification (5.14)
				FGF19				Amplification (6.65)
				FGF3				Amplification (5.6)
				CDKN2A				Deletion (0.39)
				CDKN2B				Deletion (0.44)
4	stomach	ACA T (30)		TP53	c.993+1G>A	splicing site	23.48	
				CCNE1				Amplification (34.24)
		LCNEC (70)	55	TP53	c.993+1G>A	splicing site	45.94	
				CCNE1				Amplification (67.75)
5	stomach	ACA T (30)		TP53	c.742C>T	p.Arg248Trp	11.35	
				ATRX				Deletion (0.9)
		LCNEC (70)	60	TP53	c.742C>T	p.Arg248Trp	69.23	
				ATRX				Deletion (0.94)
6	Stomach	ACA M (35)		TP53	c.673-2A>G	Splicing site	22.13	
				ATRX				Deletion (0.9)
		NETG3 (65)	30	TP53	c.673-2A>G	Splicing site	49.71	
				MYC				Amplification (5.67)
				TERT				Amplification (8.22)
7	Stomach	ACA T (30)		ATRX				Deletion (0.83)
		NETG3 (70)	60	ATRX				Deletion (0.63)
8	Stomach	ACA M (60)		TP53	c.827C>G	p.Ala276Gly	19.92	
		SCNEC (40)	60	TP53	c.827C>G	p.Ala276Gly	27.48	
9	Stomach	ACA T (40)		PIK3CA	c.2176G>A	p.Glu726Lys	17.64	
		LCNEC (60)	40	CTNNB1	c.94G>C	p.Asp32His	39.35	
				CDKN2A				Deletion (0.78)
				CDKN2B				Deletion (0.04)
10	Anus	SA,HGD (40)		MYC				Amplification (16.72)
				CDKN2A				Deletion (0)
				CDKN2B				Deletion (0)
				NBN				Deletion (0.43)
				ATRX				Deletion (0.67)
		LCNEC (60)	60	MYC				Amplification (7.035)
				CDKN2A				Deletion (0)
				CDKN2B				Deletion (0)
				RAD50				Deletion (0.7)
11	Rectum	TA, HGD (40)		PIK3CA	c.353G>A	p.Gly118Asp	3.59	
				ATRX				Deletion (0.57)
		LCNEC (60)	40	PIK3CA	c.353G>A	p.Gly118Asp	6.72	
				ATRX				Deletion (0.42)

AA, amino acid; ACA, adenocarcinoma; CNV, copy number variation; HGD, high-grade dysplasia; LCNEC, large cell neuroendocrine carcinoma; M, mucinous; NEN, neuroendocrine tumor component; NETG3, neuroendocrine tumor grade3; T, tubular; TA, tubular adenoma; SA, serrated adenoma; SCNEC, small cell neuroendocrine carcinoma.

**Table 3:** Mutational analyses of primary and metastatic MiNENs.

Patient	Site	Histology (% of component)	Ki-67 Index of NEN (%)	Gene	Code change	AA change	Allelic frequency (%)	CNV (copy number)
3	Stomach	ACA T (60)		ARID1A	c.1435C>T	p.Gln479Ter	30.62	
				TP53	c.527G>A	p.Cys176Tyr	22.64	
	LN	ACA T (100)		ARID1A	c.1435C>T	p.Gln479Ter	18.77	
				TP53	c.527G>A	p.Cys176Tyr	10.37	
				KRAS				Amplification (13.72)
5	Stomach	LCNEC (70)	60	TP53	c.742C>T	p.Arg248Trp	69.23	
				ATRX				Deletion (0.94)
	LN	LCNEC (100)	60	TP53	c.742C>T	p.Arg248Trp	62.13	
				ATRX				Deletion (0.91)
	Liver	LCNEC (100)	60	TP53	c.742C>T	p.Arg248Trp	80.22	
				ATRX				Deletion (0.98)
6	Stomach	NETG3 (65)	30	TP53	c.673-2A>G	splicing site	49.71	
				TERT				Amplification (8.22)
				RB1				Deletion (0.37)
				MYC				Amplification (5.67)
	LN	NETG3 (100)	20	TP53	c.673-2A>G	splicing site	69.3	
				TERT				Amplification (9.25)
				RB1				Deletion (0)
				AKT3				Amplification (4.93)
				RICTOR				Amplification (8.65)
				FGFR1				Amplification (6.5)
9	Stomach	LCNEC (60)	40	CTNNB1	c.94G>C	p.Asp32His	39.35	
				CDKN2A				Deletion (0.78)
				CDKN2B				Deletion (0.04)
	LN	LCNEC (100)	40	CTNNB1	c.94G>C	p.Asp32His	38.5	
				CDKN2A				Deletion (0.53)
				CDKN2B				Deletion (0)
10	Anus	LCNEC (60)	60	MYC				Amplification (70.35)
				CDKN2A				Deletion (0)
				CDKN2B				Deletion (0)
				RAD50				Deletion (0.7)
	Liver	*NET G3(100)	20	MYC				Amplification (97.12)
				CDKN2A				Deletion (0)
				CDKN2B				Deletion (0)
				FANCD2				Deletion (0.67)

*Neuroendocrine cells in a biopsy sample of a hepatic lesion did not show prominent nucleoli or necrosis, which were present in the primary large cell neuroendocrine carcinoma. Considering that the biopsy was too small to explore the full specimen, we diagnosed the hepatic lesion as a neuroendocrine tumor G3.

AA, amino acid; ACA, adenocarcinoma; CNV, copy number variation; HGD, high-grade dysplasia; LCNEC, large cell neuroendocrine carcinoma; NEN, neuroendocrine tumor component; NETG3, neuroendocrine tumor grad 3; T, tubular.

Overall, NENs carried 1.5 times more genetic variations than nNENs, most of which were CNVs. The NENs displayed frequent copy number gains for the MYC gene and copy number losses for the CDKN2A and CDKN2B genes. NENs displayed exclusive CNVs for the RB1, RAD50, FANCD2, TERT, CCND, FGF19, FGF3, FGFR1, and RICTOR genes. nNENs carried more missense mutations of the ARID1A and PIK3CA genes than NENs. In addition, nNENs carried three exclusive variants of the NBN, KRAS, and CTNNB1 genes that were not detected in NENs.

### Molecular Comparison of Primary and Metastatic GI MiNENs

To compare the characteristics of the primary and metastatic MiNENs, 11 samples from five MiNEN cases (five primary tumors and six metastatic tumors) were examined using NGS (**Table 3**). The metastatic tumors were localized in regional lymph nodes (n=4) or the liver (n=2). Histologic analyses revealed that all metastatic tumors matched the major tumor components of the primary MiNENs. The metastatic tumors retained most of the genetic variants found in the original tumors, but some cases had additional CNVs, including copy number gains for the KRAS, AKT3, RICTOR, FGFR, and FANCD2 genes, and copy number losses for the MYC and RAD50 genes.

### Molecular Differences Based on Pathologic Types and Grades of the GI MiNENs

The NENs were histologically classified as LCNEC, NETG3, or SCNEC. The LCNEC and NETG3 samples shared a number of molecular characteristics and presented many CNVs and missense mutations. By contrast, the SCNEC samples carried limited mutations in the TP53 and RB1 genes only. The nNENs were classified as adenocarcinomas or adenomas. The adenocarcinomas comprised tubular (80%) and mucinous (20%) subtypes and generally shared variants of the TP53, ATRX, and CCNE genes. Adenomas carried ATRX deletions but did not carry variants of the TP53 or CCNE1 genes.

## Discussion

This study examined the clinicopathologic and molecular characteristics of GI MiNENs. At the time of diagnosis, the majority of the MiNENs examined were advanced stage (III/IV) and frequent nodal or distant metastasis was observed, even for low tumor stages. Histologically, the metastatic tumors matched the major tumor component of the primary MiNENs. Primary MiNENs that had NEN as the major component showed a 2-fold higher rate of nodal metastasis than those with nNEN as the major component. All distant metastatic tumors were NENs. In general, NENs carried 1.5 times more mutations and CNVs than nNENs. In addition, compared with the nNENs, the NEN components carried a higher allele imbalance and displayed a more aggressive nodal metastasis, suggesting that a high allelic imbalance may be a characteristic of aggressive NEN disease behavior.

Previous studies have reported that nNENs display a closer developmental relationship with MiNENs than NECs based on comparative analyses of their pure counterparts (5). Analyses of gastro-enteric and pancreatic MiNENs have shown that mutations that are shared between NENs and nNENs usually involve cancer driver genes such as TP53, KRAS, BRAF, APC, and PI3KCA, and have higher allele frequencies (5, 9). In particular, TP53 mutation and loss of heterozygosity are the most common alterations seen in both the NEN and nNEN components of MiNENs (9, 10). Some MiNEN cases reportedly harbor cancer-related pathogenic mutations that are restricted to the NEN component, suggesting a monoclonal origin and a multistep progression model of gastric MiNEN development (8, 10, 11). Sun et al. reported that up to 50% of gastric MiNENs displayed CCNE1 gene amplification and increased protein expression (12). Here, we identified frequent TP53 gene mutations (18/22, 81.8%) and limited CCNE1 gene amplification (4/22, 18.2%) in gastric MiNENs. We did not detect mutations of the BRAF, APC, or SMAD4 genes in the adenocarcinomas, although these genes have been reported as frequently mutated in previous studies. We identified frequent CNVs of the ATRX and RB1 genes in NENs and key driver mutations of the ARID1A, PIK3CA, CTNNB1, and MYC genes in nNENs, especially adenocarcinomas. Overall, our findings show that NENs and nNENs both display pathogenic mutations, most of which are in genes involved in chromatin remodeling pathways.

Molecular subdivision, including neuroendocrine tumors (NETs) and neuroendocrine carcinomas (NECs), has been established *via* differential mutations of ATRX/DAXX/MEN1 and TP53/RB1 in the pancreas (13). The molecular criteria have not been established for GI MiNENs since the molecular landscape has been suggested to pure adenocarcinomas and ATRX mutation has not been reported (5). ATRX is a novel tumor suppressor gene that encodes an SWI/SNF-like chromatin remodeling protein (11). ATRX deletion mutations occur in various malignancies, including glial tumors, pediatric adrenocortical carcinoma, osteosarcoma, and neuroblastoma (3).

Here, we identified ATRX mutations (n=10/27, 37.0%), primarily partial loss in GI MiNENs. A recent study suggests that ATRX haploinsufficiency cooperates with p53 deficiency to promote multiple types of carcinoma and sarcoma, including epithelioid sarcoma, angiosarcoma, undifferentiated pleomorphic sarcoma, papillary serous carcinoma, biliary carcinoma, malignant peripheral nerve sheath tumor, and high-grade glioma (14). Our study identified ten samples carrying a partial ATRX loss, of which seven samples carried additional mutations of the tumor suppressor TP53 (n=5/7, 71.4%) or the oncogenic PIK3CA (n=2/7, 28.6%). Despite the apparent genetic difference between pancreatic NETs and NECs, NETG3 and LCNEC were comparable in this study. In addition, although ATRX deletion was frequently observed in NETG3s and LCNECs, it was not seen in SCNECs or nNENs. SCNECs carried mutations of the RB1 and TP53 loci; these are frequently observed mutation sites, validated by prior studies (8, 15, 16). Further studies with a large number of cases will be conducted to evaluate the presence and functional significance of ATRX deletion in GI MiNENs.

In the cases examined here, nNENs were usually located superficially and NENs were located in a deeper area of the GI tract, which was considered the invasive front. We hypothesized that NENs might develop from superficial nNENs *via* the accumulation of additional molecular aberrations. For 10 of the 11 (90.9%) pairs of nNENs and NENs examined here, the two components of the MiNEN shared common genetic alterations. However, 3 of these 10 cases carried additional mutations in the NEN and nNEN components. Based on these findings, we suggest that MiNENs are primarily of monoclonal origin, but can undergo bi-phenotypic differentiation during the carcinogenesis process in some cases (4). We also identified one case of gastric MiNEN that had no shared molecular alterations in the nNEN and NEN components, indicating that MiNEN can emerge from two distinct entities during tumorigenesis in some cases.

This study has some limitations that need to be further improved. First, the study cohort was relatively small and consisted mainly of gastric MiNEN, thus, it was difficult to generalize the results for gastrointestinal MiNEN in general. Second, genetic evaluation was limited by selected genes in a commercial panel. Lastly, all sequencing data in this study were extracted from FFPE blocks; it is known that formalin-fixation would cause nucleic acid fragmentation, degradation, and cross-linking to proteins (17).

In conclusion, this study used NGS to compare the molecular characteristics of GI MiNENs that were classified according to tumor component and grade. The majority of pathogenic variants identified were shared between the paired NEN and nNEN components of the MiNENs. However, some NEN and nNEN samples carried distinct mutations that were not seen in the corresponding paired component. Metastatic tumors retained most of the genetic variants found in the primary MiNENs, but also gained additional copy number alterations. Our results give clues to the mechanisms underlying the developmental progression of gastric MiNENs. Further studies involving large numbers of cases and additional functional studies should be performed to clarify the detailed molecular characteristics and tumorigenic mechanisms of GI MiNENs.

## Data Availability Statement

The datasets presented in this study can be found in online repositories. The names of the repository/repositories and accession number(s) can be found in the article/**Supplementary Material**.

## Ethics Statement

The studies involving human participants were reviewed and approved by Institutional Review Board at the Chungnam National University Hospital, Daejeon, South Korea (2019-11-043). Written informed consent for participation was not required for this study in accordance with the national legislation and the institutional requirements.

## Author Contributions

GB, NY and M-KY had full access to all of the data in the study and take responsibility for the integrity of the data and the accuracy of the data analysis. Study concept and design, M-KY. Acquisition, analysis, and interpretation of data, GB and NY. Drafting of the manuscript, GB. Critical revision of the manuscript for important intellectual content, GB, NY and M-KY. Obtained funding, GB, NY and M-KY. Administrative technical support, NY. Study supervision, GB, NY and M-KY. All authors contributed to the article and approved the submitted version.

## Funding

This work was supported by the National Research Foundation of Korea (NRF) grant funded by the Korea government (MSIT) (No. 2019R1G1A1100578), a grant of Korea Health Technology R&D Project through the Korea Health Industry Development Institute (KHIDI) funded by the Ministry of Health & Welfare, Republic of Korea (grant number: HR20C0025), and a grant of Translational R&D Project through the Institute for Bio-Medical Convergence, Incheon St. Mary’s Hospital, The Catholic University of Korea.

## Conflict of Interest

The authors declare that the research was conducted in the absence of any commercial or financial relationships that could be construed as a potential conflict of interest.

## Publisher’s Note

All claims expressed in this article are solely those of the authors and do not necessarily represent those of their affiliated organizations, or those of the publisher, the editors and the reviewers. Any product that may be evaluated in this article, or claim that may be made by its manufacturer, is not guaranteed or endorsed by the publisher.

## Supplementary Material

Supplementary Table 1 Gene lists for Oncomine Comprehensive Assay version 3 (n=161).
