# Supplementary material for: Clinicopathologic and Molecular Characteristics of Gastrointestinal MiNENs
Source: Front Oncol. 2021 Aug 4;11:709097. doi: 10.3389/fonc.2021.709097 (PMC8371704; doi:10.3389/fonc.2021.709097)
Supplement: Supplementary Table 1 — Gene lists for Oncomine Comprehensive Assay version 3 (n=161). [file Table_1.docx]

Gene lists for Oncomine Comprehensive Assay version 3 (n=161)

| Small nucleotide variant and indels (Hotspot genes, n=86, from DNA) | | | | |  |
| --- | --- | --- | --- | --- | --- |
| *AKT1* | *ERBB4* | *IDH2* | *MTOR* | *SPOP* | *MDM4* |
| *ALK* | *ESR1* | *JAK1* | *MYD88* | *SRC* | *MYC* |
| *AR* | *EZH2* | *JAK2* | *NFE2L2* | *STAT3* | *MYCN* |
| *ARAF* | *FGFR1* | *JAK3* | *NRAS* | *U2AF1* | *NTRK1* |
| *BRAF* | *FGFR2* | *KDR* | *PDGFRA* | *XPO1* | *NTRK2* |
| *BTK* | *FGFR3* | *KIT* | *PIK3CA* | *AKT2* | *PDGFRB* |
| *CBL* | *FLT3* | *KNSTRN* | *PPP2R1A* | *AKT3* | *PIK3CB* |
| *CDK4* | *FOXL2* | *KRAS* | *PTPN11* | *AXL* | *ROS1* |
| *CHEK2* | *GATA2* | *MAGOH* | *RAC1* | *CCND1* | *SMAD4* |
| *CSF1R* | *GNA11* | *MAP2K1* | *RAF1* | *CDK6* | *TERT* |
| *CTNNB1* | *GNAQ* | *MAP2K2* | *RET* | *ERCC2* | *TOP1* |
| *DDR2* | *GNAS* | *MAPK1* | *RHEB* | *FGFR4* |  |
| *EGFR* | *HNF1A* | *MAX* | *RHOA* | *H3F3A* |  |
| *ERBB2* | *HRAS* | *MED12* | *SF3B1* | *HIST1H3B* |  |
| *ERB83* | *IDH1* | *MET* | *SMO* | *MAP2K4* |  |
| Full-length genes (n=48, from DNA) | | | |  |  |
| *ATM* | *NF2* | *TP53* | *CDKN2B* | *MSH6* | *RAD51* |
| *BAP1* | *NOTCH1* | *TSC1* | *CHEK1* | *NBN* | *RAD51B* |
| *BRCA1* | *PIK3R1* | *TSC2* | *CREBBP* | *NOTCH2* | *RAD51C* |
| *BRCA2* | *PTCH1* | *ARID1A* | *FANCA* | *NOTCH3* | *RAD51D* |
| *CDKN2A* | *PTEN* | *ATR* | *FANCD2* | *PALB2* | *RNF43* |
| *FBXW7* | *RB1* | *ATRX* | *FANCI* | *PMS2* | *SETD2* |
| *MSH2* | *SMARCB1* | *CDK12* | *MLH1* | *POLE* | *SLX4* |
| *NF1* | *STK11* | *CDKN1B* | *MRE11A* | *RAD50* | *SMARCA4* |
| Copy number variation (n=47, from DNA) | | | |  |  |
| *AKT1* | *FGFR1* | *MDM2* | *PPARG* | *CCND3* | *NTRK2* |
| *AR* | *FGFR2* | *MDM4* | *TERT* | *CDK2* | *NTRK3* |
| *CCND1* | *FGFR3* | *MET* | *AKT2* | *CDKN2A* | *PDGFRB* |
| *CCNE1* | *FGFR4* | *MYC* | *AKT3* | *CDKN2B* | *PIK3CB* |
| *CDK4* | *FLT3* | *MYCL* | *ALK* | *ESR1* | *RICTOR* |
| *CDK6* | *IGF1R* | *MYCN* | *AXL* | *FGF19* | *TSC1* |
| *EGFR* | *KIT* | *PDGFRA* | *BRAF* | *FGF3* | *TSC2* |
| *ERBB2* | *KRAS* | *PIK3CA* | *CCND2* | *NTRK1* |  |
| Fusions and splice variants (n=51 from RNA) | | | |  |  |
| *ALK* | *FGFR1* | *ROS1* | *FLT3* | *NOTCH4* | *RAD51B* |
| *AXL* | *FGFR2* | *AKT2* | *JAK2* | *NRG1* | *RB1* |
| *BRAF* | *FGFR3* | *AR* | *KRAS* | *NTRK2* | *RELA* |
| *EGFR* | *NTRK1* | *BRCA1* | *MDM4* | *NUTM1* | *RSPO2* |
| *ERBB2* | *NTRK3* | *BRCA2* | *MET* | *PDGFRB* | *RSPO3* |
| *ERG* | *PDGFRA* | *CDKN2A* | *MYB* | *PIK3CA* | *TERT* |
| *ETV1* | *PPARG* | *ERBB4* | *MYBL1* | *PRKACA* |  |
| *ETV4* | *RAF1* | *ESR1* | *NF1* | *PRKACB* |  |
| *ETV5* | *RET* | *FGR* | *NOTCH1* | *PTEN* |  |
